# Resistance of *Leishmania (Leishmania) amazonensis *and *Leishmania (Viannia) braziliensis *to nitric oxide correlates with disease severity in Tegumentary Leishmaniasis

**DOI:** 10.1186/1471-2334-7-7

**Published:** 2007-02-22

**Authors:** Angela Giudice, Ilza Camada, Paulo TG Leopoldo, Júlia MB Pereira, Lee W Riley, Mary E Wilson, John L Ho, Amelia Ribeiro de Jesus, Edgar M Carvalho, Roque P Almeida

**Affiliations:** 1 Serviço de Imunologia, Hospital Universitário Prof. Edgard Santos, Instituto de Investigação em Imunologia (iii), Universidade Federal da Bahia, Salvador, Bahia, Brazil; 2 Departamento de Fisiologia, Universidade Federal de Sergipe, Aracaju, Sergipe, Brazil; 3 Division of Infectious Diseases, School of Public Health, University of California, Berkeley, CA, USA; 4 Department of Internal Medicine, Microbiology, University of Iowa College of Medicine, Iowa City, IA, USA; 5 Department of Medicine, Division of International Medicine and Infectious Diseases, Weill Medical College of Cornell University, New York, NY, USA

## Abstract

**Background:**

Nitric oxide (NO^•^) plays a pivotal role as a leishmanicidal agent in mouse macrophages. NO^• ^resistant *Escherichia coli *and *Mycobacterium tuberculosis *have been associated with a severe outcome of these diseases.

**Methods:**

In this study we evaluated the *in vitro *toxicity of nitric oxide for the promastigote stages of *Leishmania (Viannia) braziliensis *and *Leishmania (Leishmania) amazonensis *parasites, and the infectivity of the amastigote stage for human macrophages. Parasites were isolated from patients with cutaneous, mucosal or disseminated leishmaniasis, and NO^• ^resistance was correlated with clinical presentation.

**Results:**

Seventeen isolates of *L. (L.) amazonensis *or *L. (V.) braziliensis *promastigotes were killed by up to 8 mM of more of NaNO_2 _(pH 5.0) and therefore were defined as nitric oxide-susceptible. In contrast, eleven isolates that survived exposure to 16 mM NaNO_2 _were defined as nitric oxide-resistant. Patients infected with nitric oxide-resistant *Leishmania *had significantly larger lesions than patients infected with nitric oxide-susceptible isolates. Furthermore, nitric oxide-resistant *L. (L.) amazonensis *and *L. (V.) braziliensis *multiplied significantly better in human macrophages than nitric oxide-susceptible isolates.

**Conclusion:**

These data suggest that nitric oxide-resistance of *Leishmania *isolates confers a survival benefit for the parasites inside the macrophage, and possibly exacerbates the clinical course of human leishmaniasis.

## Background

Leishmaniasis is a parasitic disease caused by the *Leishmania *spp. protozoa, transmitted to the skin of a mammalian host during the bite of an infected female sand fly vector. Infections range in severity from asymptomatic to disfiguring forms of tegumentary and potentially fatal visceral leishmaniasis [1,2]. American tegumentary leishmaniasis (ATL) presents a spectrum of clinical manifestations characterized by cutaneous (CL), mucosal (ML), disseminated (DL) and diffuse cutaneous leishmaniasis (DCL). The major *Leishmania *species that cause ATL in the New World are *L*. (*V.*) *braziliensis*, *L*. (*V.*) *guyanensis*, *L*. (*L.*) *amazonensis *and *L*. (*L.*)*mexicana*. The typical clinical manifestation of American CL is a single ulcerated lesion with elevated borders, frequently located on the inferior limbs [3]. Mucosal leishmaniasis (ML) is a destructive disease that predominantly affects the nasopharyngeal mucosa. The disease is most common in areas of *L. (V.) braziliensis *transmission and usually occurs months or years after cutaneous leishmaniasis [4]. Disseminated leishmaniasis (DL) has been reported almost exclusively in northern and northeastern Brazil. DL is characterized by the appearance of multiple pleomorphic lesions in two or more noncontiguous areas of the body [5].

*Leishmania *is a digenetic protozoan with an extracellular flagellated promastigotes form which replicates and matures to the infectious metacyclic form in the gut of the sand fly vector. The promastigotes is transmitted to a mammalian host during the bite of an infected sand fly. Promastigotes undergo facilitated phagocytosis by a macrophage and convert to the obligate intracellular amastigote life stage [6,7]. Amastigotes survive in macrophage phagolysosomes, a hostile environment for many microbes. *Leishmania *spp. must undergo profound biochemical and morphological adaptations to survive successfully and multiply within macrophages [7]. The mechanisms through which the parasite resists killing within the toxic environment of the phagolysosome remain incompletely defined.

Leishmaniasis is controlled through cell-mediated immune defenses [8]. Murine models have illustrated that macrophages produce IL-12, which induces CD4+ T cells and NK cells to release interferon gamma (IFN-γ), polarizing the immune response toward a type 1 (Th1 type) phenotype [9,10]. In murine systems, IFN-γ has been shown to synergize with another macrophage derived cytokine, tumor necrosis factor alpha (TNF-α), activating nitric oxide synthase (iNOS or NOS2) to produce nitric oxide (NO^•^) with resultant in killing of intracellular parasites [11-13]. NO^• ^is generated from the oxidation of the terminal guanidine nitrogen atoms of the L-arginine by NADPH dependent enzyme nitric oxide synthase (NOS) [14]. In murine models of leishmaniasis, NO^•^-dependent mechanisms have been shown to be critical for control of *Leishmania *infection[15,16]. The role of NO^•^ in leishmanicidal activity of human macrophages, has been debated [17]. However, recent data suggest that NO^• ^plays a role in the response of human macrophages to intracellular infections, but the nature of this role is not yet clear [18,19].

Putative NO^•^-mediated leishmanicidal actions in eukaryotic cells include inhibition of mitochondrial respiration, inactivation of peroxidases, increasing susceptibility to oxidant damage, inhibition of glycolysis, mutation of DNA, inhibition of DNA repair and synthesis, S-nitrosylation, ADP-ribosylation, tyrosine nitration of proteins, disruption of Fe-S clusters, zinc fingers or heme groups, and peroxidation of membrane lipids [20,21]. The *Leishmania *spp. possesses unique antioxidant mechanisms and enzymes. Notably, they convert their abundant GSH stores to trypanothione (TSH) and use TSH reductase/oxidase systems for redox cycling [22]. The protozoa express an iron superoxide dismutase (SOD) but not a manganese SOD, and they have peroxidoxins for handling oxidative stress [23-26]. Nonetheless, oxidant resistance in these parasites are inducible [27], and one expects these systems are susceptible to inactivation by oxidant species similar to other eukaryotes.

Resistance to nitric oxide has been described in *E. coli *and *M. tuberculosis*. Resistant isolates have been associated with a more severe outcome of disease than that caused by non-resistant strains [28]. However, natural NO^• ^resistance in *Leishmania *spp. isolates has not previously been described. In the present study, we evaluated the effect of NO^• ^generated from NaNO_2 _(pH 5.0) on the viability of *L. (V.) braziliensis *and *L. (L.) amazonensis *promastigotes. NO^• ^resistant *Leishmania *amastigotes multiplied significantly better than nitric oxide-susceptible parasites during infection of human macrophages. Furthermore, NO^• ^resistance was directly associated with lesion size, a clinical measure of disease severity.

## Methods

### Parasites

*L. (L.) amazonensis *and *L. (V.) braziliensis *parasites were obtained by needle aspiration of lesions from patients with CL, DL or ML. Parasites for study were randomly selected from frozen nitrogen *Leishmania *stocks by investigators blinded to the *Leishmania *species or clinical form of leishmaniasis. Parasites were speciated by isoenzyme electrophoresis and monoclonal antibodies as described. This analysis was performed by Departamento de Bioquimica e Biologia Molecular, Instituto Oswaldo Cruz, FIOCRUZ, Rio de Janeiro, Brazil [29].

### Isolation and cultivation of *L. (V.) braziliensis *and *L. (L.) amazonensis*

Parasite isolates *L. (V.) braziliensis *(n = 17) and *L. (L.) amazonensis *(n = 11) were initially cultivated from patient specimens in tubes with biphasic medium (NNN) consisting of rabbit blood agar overlaid with liver infusion tryptose (LIT), supplemented with 10% heat inactivated fetal bovine serum medium (Sigma Chemical Co., St. Louis, MO). Following isolation, parasite isolates were cryopreserved. The time of storage of the selected strains was similar (p > 0.05). The parasites selected for study had not been previously passaged in liquid culture medium before the beginning of the present study. After selection, parasite isolates were expanded in Schneider's insect medium (Sigma) pH 7.2 supplemented with 10% fetal bovine serum (FBS) and 2% male human urine at 25°C (complete Schneider medium).

### Promastigote NO^• ^susceptibility assays

#### Thymidine incorporation

*L. (L.) amazonensis *(n = 10) and *L. (V.) braziliensis *(n = 6) promastigotes in log phase growth were adjusted to 2 × 10^7 ^parasites/ml in Hanks' balanced solution (HBBS Sigma, pH 5.0). Twenty μl aliquots containing 4 × 10^5 ^parasites were placed in 96-well U shaped plates containing 180 μl of 0 to 16 mM NaNO_2 _(freshly prepared NO^•^ donor) in Hanks Balanced Solution, pH 5.0. After 4 hours incubation at 25°C, plates were centrifuged (700 × g for 10 minutes). The viability of the remaining parasites was assessed by incubation for 20 hr in 200 μl of complete Schneider's medium with 1 μCi of Thymidine (^3^H-TdR; ICN Immunochemicals, Costa Mesa, CA, USA) to allow them to enter logarithmic growth. Thymidine incorporation was assessed on a β counter.

#### MTT assay

*L. (L.) amazonensis *(n = 5) and *L. (V.) braziliensis *promastigotes (n = 14) in log phase growth were adjusted to 5 × 10^7 ^parasites/ml in Hanks' balanced solution (HBBS Sigma, pH 5.0). Twenty-μl aliquots were incubated in 180 μl of 0 to 16 mM NaNO_2_(freshly prepared NO^•^ donor) in Hanks Balanced Solution, pH 5.0 in 96-well U shaped plates. After 4 hrs incubation at 25°C, plates were centrifuged (700 × g for 10 minutes) and parasites were resuspended with 200 μl of complete Schneider medium. After an additional 20 hrs at 25°C and centrifugation, parasite viability was measured by incubation in 0.5 mg/ml of MTT [3-(4,5-dimetthythiazol-2-yl)-2,5-diphenyltetiazolium bromide] in Hanks solution, pH 7.0 at 25°C for 4 hrs, followed by dilution in an equal volume 0.04 N HCl in isopropanol. Living mitochondria convert MTT to dark blue formazan that is soluble in acid-isopropanol and detectable on a microplate reader at 540 nm. The percentage of viability was calculated from the OD ratio of untreated versus NO^•^-treated parasites × 100 [27]. For each parasite isolate 3 experiments at least were performed to test for NO^•^ susceptibility. The thymidine incorporation and MTT assays were done with 28 *Leishmania *isolates of both species. Seven isolates were tested with both methods.

The virulence of *Leishmania *spp. is highest in stationary phase, or metacyclic organisms. Nonetheless the MTT and [^3^H]-TdR uptake assays are most sensitive for log phase organisms. We previously reported that these assays of virulence and oxidant sensitivity in log phase correlate with oxidant sensitivity and virulence in stationary phase organisms [27,30]. As such, viability assays were performed using log phase promastigotes, whereas studies of interactions with mammalian cells utilized stationary phase organisms.

### Macrophage cultures

Peripheral blood mononuclear cells (PBMCs) were isolated from the peripheral blood of three different healthy human donors. Briefly, heparinized blood was diluted 1:2 with 0.15 M NaCl and overlaid on Ficoll Hypaque (LSM; Organon Teknika corporation, Durham, NC, USA). After centrifugation, mononuclear cells were collected at the plasma – Ficoll interface, washed three times and resuspended in RPMI 1640 with 10% heat inactivated human AB serum, 100 U penicillin/ml and 100 μg streptomycin/ml (complete medium) (GIBCO BRL, Grand Island, NY). Monocytes were separated from 1 × 10^6 ^PBMCs by adherence to 8 well Lab Tek plates for 2 h at 37°C, 5% CO_2_, non-adherent cells were removed by washing, and complete medium was added. Adherent monocytes differentiated to macrophages over six days incubation at 37°C in 5% CO_2_.

### Macrophage infection

One NO^•^-resistant and one NO^•^-susceptible isolate each of *L. (V.) braziliensis *and *L. (L.) amazonensis *(total 4 isolates)were selected for the macrophage infection assays. Three to 4 replicate assays were performed for each isolate. Promastigotes were maintained at 25°C in Schneider's insect medium (Sigma) pH 7.2 supplemented with 10% fetal bovine serum (FBS) and 2% human male urine at 25°C (complete Schneider's medium). Promastigotes in stationary- phase of growth were used in all experiments. All experiments were performed in 3 assays for *L. (V.) braziliensis *and 4 assays for *L. (L.) amazonensis *using PBMC/macrophages from 3 different healthy volunteers. The same donors were used for the different species so that the results are directly comparable. After Six-day monocyte-derived macrophages were infected with a 10:1 ratio of stationary – phase promastigotes to macrophages for 2 hours at 35°C, 5% CO_2_. Extracellular parasites were removed by gentle washing and infected macrophages were maintained for up to 96 h. Cells were stained with Giemsa and the infection levels were enumerated microscopically by counting the infected cells and parasites per 100 macrophages by three independent observers, blinded to the experimental conditions.

### Epidemiological and clinical evaluations

Clinical characteristics of the patients such as age, lesion size, Montenegro skin reaction, duration of disease and clinical manifestation of leishmaniasis were determined from clinical records after characterizing the NO^• ^susceptibility of isolates. Adequate data were available for fourteen patients. Most patients were identified and diagnosed at the Corte de Pedra Health Post, located in an endemic area for cutaneous leishmaniasis situated in the southeast of the state of Bahia, Brazil. The remainder of patients was referred to the University Hospital Prof. Edgard Santos of the Federal University of Bahia, Brazil. This study was approved by the Ethical Committee of the Hospital Universitário Prof. Edgard Santos and an informed consent was obtained from all participants or their parents or guardians if patients were less than 18 years old.

### Statistical analysis

Student's t-test was used to compare the age, lesion appearance, and human macrophage infection studies. Lesion size and Montenegro diameter were analyzed by Mann-Whitney nonparametric test. Fischer's Exact test was used to compare NO^•^-resistant versus NO^•^-susceptible *L. (L.) amazonensis *and *L. (V.) braziliensis*. An alpha of 5% (p ≤ 0.05) was considered for statistical significance (two tailed). Statistical analysis was performed using GraphPad Prism 3.0 (GraphPad software, San Diego, CA, USA).

## Results

### Evaluation of *Leishmania spp*. promastigotes resistance to NO^• ^and correlation with clinical disease

**Figure 1:**
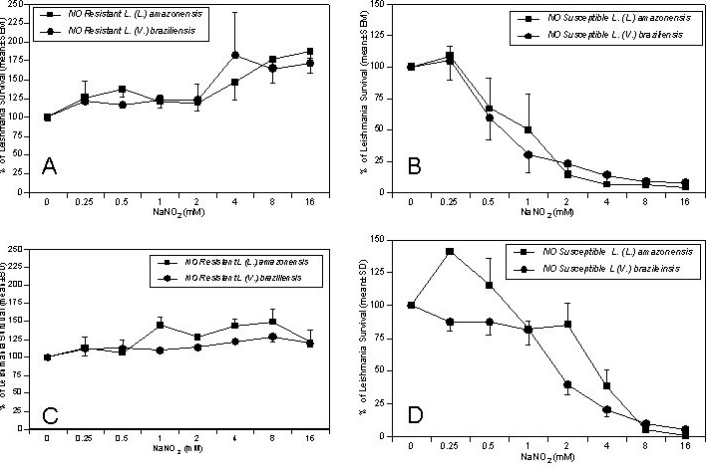
**Survival of *L. (V.) braziliensis *and *L.(L.) amazonensis *promastigotes in increasing concentrations of NO^• ^measured by Thymidine incorporation (A and B) and colorimetric MTT assay (C and D)**. Promastigotes in HBSS pH 5.0 were exposed to increasing NaNO_2 _concentrations (0.25–16 mM) for 4 hours. Viability was measured by incorporation of ^3^H-TdR or conversion of MTT of formazan as described in the Methods section. Data in panels A and B represent mean ± SEM percentage survival for (**1A**) 8 resistant *L. (L.) amazonensis *and two resistant *L. (V.) braziliensis *isolates, **(1B) **two susceptible *L. (L.) amazonensis *and 4 susceptible *L. (V.) braziliensis *isolates as measured by thymidine incorporation. Data in panels C and D represent the mean ± SEM MTT conversion for (**1C**) 3 NO^•^- resistant *L. (L.) amazonensis *and 2 NO^•^-resistant *L. (V.) braziliensis *isolates, or **(1D) **2 susceptible *L. (L.) amazonensis *and 12 susceptible *L. (V.) braziliensis *isolates.

The susceptibility of *L. (L) amazonensis *(n = 11) and *L. (V) braziliensis *(n = 17) promastigotes to NO^•^ toxicity was evaluated using two measures of parasite viability: first, the rate of [^3^H]-thymidine incorporation into parasite DNA, and second, a colorimetric measure of mitochondrial activity according to MTT metabolism to formazan. Our preliminary titrations led us to a definition of NO^• ^susceptibility as measured viability that is less than 5% of control parasites after exposure to 8 mM NaNO_2_. Using this definition, we found that 73% (8 of 11) of the *L. (L.) amazonensis *isolates and 18% (3 of 17) *L. (V.) braziliensis *isolates were resistant to NO^•^ (Table 1). Of the NO^•^-susceptible isolates, a titration of NaNO_2 _from 0.25 mM to 16 mM showed concentration-dependent killing of susceptible isolates with nearly 100% killing at 8 mM NaNO_2_. In contrast, NO^•^-resistant *L. (L.) amazonensis *and *L. (V.) braziliensis *isolates remained viable even in 16 mM NaNO_2 _(Figure 1, Tables 2, 3, 4 and 5). The storage time in liquid nitrogen of the NO^•^-susceptible isolates (mean ± SD = 6.6 ± 3.2 years) was similar to the NO^• ^resistant isolates (7.8 ± 2.7 years), p = 0.3.

**Table 1:** Relation between *Leishmania* species and NO-resistance

Isolates	Susceptible	Resistant	Total
*L. amazonensis*	3	8 (73%)	11
*L. braziliensis*	14	3 (18%)	17
Total	17	11	28

Differences between the proportions were statistically significant (Fisher Exact Test, p < 0.006)

**Table 2:** Thymidine incorporation assay of resistant *L. (L.) amazonensis and L. (V.) braziliensis *promastigotes to NO (NaNO_2_) donor *in vitro*.

***L (L.) amazonensis***	NaNO_2 _Concentration (mM)							
	
	0	0.25	0.5	1	2	4	8	16
10184	1804	2205	3077	2373	1284	1426	1648	1435
436	1683	1928	2189	2544	2704	3755	4366	4047
10432	3874	4240	4285	3914	3453	3312	2880	2450
10048	826	1008	1167	1150	1243	1159	1114	1549
10076	1644	2094	2870	2090	1652	1860	3874	3817
484077	788	988	928	961	1009	1158	873	1674
10047	5765	7551	5789	4416	9139	7024	19181	17839
AC	5995	10034	9731	7218	6162	16305	10955	11195

***L. (V.) braziliensis***	NaNO_2 _Concentration (mM)							
	
	0	0.25	0.5	1	2	4	8	16

13314	1626	2407	1829	1923	2340	3902	2950	2677
H.R	1958	1865	2367	2549	1993	2442	2909	3511

**Table 3:** Thymidine incorporation assay of susceptible *L (L.) amazonensis*

***L (L.) amazonensis***	NaNO_2 _Concentration (mM)							
	
	0	0.25	0.5	1	2	4	8	16
8653	1227	1244	513	262	174	102	81	61
9667	1364	1595	1255	1078	200	65	75	54

***L. (V.) braziliensis***	NaNO_2 _Concentration (mM)							
	
	0	0.25	0.5	1	2	4	8	16

13323	2339	3217	1636	215	92	88	76	83
Lb-001	2213	2368	1978	1319	830	482	268	242
13352	1879	1227	1318	870	868	474	326	196
13468	1626	1796	169	78	73	85	89	97

4 × 10^5 ^*L. (L.) amazonensis *and *L. (V.) braziliensis *promastigotes in HBSS pH 5.0 were exposed to increased NaNO_2 _concentrations (0.25–16 mM) for 4 hours. The *Leishmania *were then washed, distributed in parasite growth medium in the presence of 1 μci/ml of ^3^H-TdR. After 20 hours of incubation, the incorporation of Thymidine was measured in Beta scintillation counter (CPM). Data are mean ± SD of NO^•^-resistant (Table 2) or NO^•^-susceptible (Table 3) isolates of *L. (L.) amazonensis *and *L. (V.) braziliensis*

**Table 4:** MTT colorimetric assay of resistant *L. (L.) amazonensis and L. (V.) braziliensis *promastigotes to NO (NaNO_2_) donor *in vitro*.

***L (L.) amazonensis***	NaNO_2 _Concentration (mM)							
	
	0	0.25	0.5	1	2	4	8	16
10184	0.558	0.678	0.729	0.693	0.726	0.989	0.824	0.518
436	0.172	0.231	0.197	0.257	0.207	0.197	0.216	0.265
10432	0.098	0.081	0.072	0.158	0.131	0.153	0.154	0.112

***L. (V.) braziliensis***	NaNO_2 _Concentration (mM)							
	
	0	0.25	0.5	1	2	4	8	16

13314	0.379	0.455	0.404	0.421	0.438	0.462	0.506	0.47
14214	0.242	0.248	0.285	0.261	0.268	0.294	0.295	0.279

**Table 5:** MTT colorimetric assay of susceptible *L (L.) amazonensis*

***L (L.) amazonensis***	NaNO_2 _Concentration (mM)							
	
	0	0.25	0.5	1	2	4	8	16
9667	0.488	0.682	0.463	0.373	0.335	0.25	0.027	0.003
9986	0.194	0.278	0.263	0.171	0.197	0.049	0.01	0.002

***L. (V.) braziliensis***	NaNO_2 _Concentration (mM)							
	
	0	0.25	0.5	1	2	4	8	16

11155	0.389	0.478	0.488	0.432	0.113	0.025	0.007	0.004
13396	0.292	0.269	0.28	0.325	0.224	0.038	0.012	0.009
13323	0.407	0.265	0.382	0.684	0.228	0.157	0.033	0.014
Lb 001	0.249	0.178	0.191	0.167	0.169	0.125	0.091	0.03
9291	0.628	0.645	0.437	0.253	0.101	0.033	0.051	0.02
13690	0.558	0.646	0.448	0.326	0.13	0.055	0.023	0.073
14183	0.178	0.166	0.168	0.096	0.048	0.035	0.017	0.009
9139	0.446	0.277	0.221	0.305	0.182	0.093	0.047	0.012
13183	0.598	0.39	0.38	0.373	0.02	0.036	0.014	0.007
13548	0.547	0.58	0.894	0.584	0.382	0.246	0.053	0.032
14349	0.812	0.556	0.426	0.404	0.208	0.108	0.085	0.086
14808	0.261	0.231	0.251	0.160	0.096	0.074	0.024	0.08

1 × 10^6 ^*L. (L.) amazonensis *and *L*. (V.) *braziliensis *promastigotes contained were exposed for 4 hours to increasing concentrations of the NO^• ^donor NaNO_2 _(0.25–16 mM) in HBSS pH 5.0. The cells were then washed and distributed in parasite growth medium for 20 hours. After this time the supernatants were removed and the parasites were incubated in HBSS, pH 7.0 plus 10 μl of MTT for 4 hours. Viability was a measured by the conversion of MTT to formazan and is expressed as the OD at 540 nm. Data are mean ± SD of NO^•^- resistant *Leishmania* isolates (Table 4) or NO^• ^susceptible *Leishmania *isolates (Table 5).

### Infection in vitro of human macrophages

**Figure 2:**
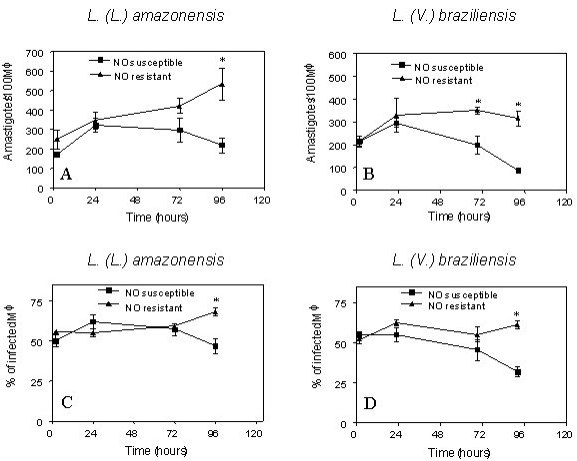
Infection of human macrophages with resistant or susceptible *L. (V.) braziliensis *or *L. (L.) amazonensis *isolates. Human Mφ from 3 healthy donors were infected with NO^•^-susceptible or NO^•^-resistant *L*. (*L.*)*amazonensis *or *L. (V.) braziliensis *and evaluated at the designated time points for the level of intracellular infection. After monolayers were stained with Giemsa, the level of infection was expressed as number of amastigotes per 100 Mφ **(A, B), **and as the percentage of infected Mφ **(C, D) **for *L. (L.) amazonensis ***(A, C) **or *L. (V.) braziliensis *isolates **(B, D)**. The data are expressed as the mean ± SEM from 3 separate experiments for *L. (V.) braziliensis *and the mean ± SEM from 4 experiments for *L. (L.) amazonensis*. Parasites were used in stationary phase of growth. Statistical analysis was performed using the paired t-test.

Two isolates each of *L. (L.) amazonensis *and *L. (V.) braziliensis*, one NO^•^-resistant and one NO^•^-susceptible were evaluated for their ability to infect and proliferate within culture-derived human macrophages *in vitro*. The data demonstrate that NO^•^-resistant and NO^•^-susceptible *L (L.) amazonensis *and *L. (V.) braziliensis *infected human macrophages with similar efficacy, as demonstrated by a similar degree of macrophage infection at 2 hours with all isolates (Figure 2, p > 0.05). Beginning 24 h after infection, intracellular macrophage killing of NO^•^-susceptible parasites of both *Leishmania *species was evident from the declining parasite numbers. In contrast, NO^•^-resistant parasites either maintained an unchanged infection level [*L. (V.) braziliensis*] or multiplied [*L. (L.) amazonensis*]. Thus, at the 96 hr time point the numbers of intracellular NO^•^-resistant *L. (L.) amazonensis *amastigotes was significantly higher than NO^•^-susceptible parasites (mean ± SD = 534 ± 164 versus 219 ± 75, p = 0.01). Similarly, the numbers of intracellular NO^•^-resistant *L. (V.) braziliensis *was significantly higher than NO^•^-susceptible parasites (mean ± SD = 315 ± 56 versus 87 ± 3, p = 0.002) (Figure 2A and 2B). When the results were calculated as the percent of cells infected, data revealed there were also significantly higher numbers of macrophages infected by NO^•^-resistant compared to NO^•^-susceptible *L. (L.) amazonensis *(mean ± SD = 68 ± 5.2 versus 47 ± 10, p = 0.008), and higher numbers of macrophages infected with NO^• ^– resistant versus susceptible *L. (V.) braziliensis *(61 ± 4 versus 32 ± 5, p = 0.002). This suggests that parasites spread to new cells in the macrophage monolayer *in vitro *(Figure 2C and 2D). Although we did not evaluate the mechanisms, these data suggest that NO^•^-resistant amastigotes survive and multiply in resting human macrophages better than susceptible isolates.

### Epidemiological and clinical evaluations

**Figure 3:**
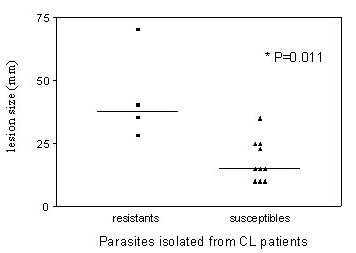
Association between NO^•^-susceptibility of the *Leishmania *isolate and size of the initial cutaneous lesion in CL patients. Patients (*n *= 14) with cutaneous leishmaniasis were assessed for lesion size at the time of clinical presentation. This is graphed with the correlating 4 NO^•^-resistant ***Leishmania *(*3 L (L.) amazonensis *and 1 *L. (V.) braziliensis ***or 10 NO^•^-susceptible ***Leishmania *(2 *L (L.) amazonensis *and 8* L (V.) brazilienis*)**, (p = 0.01, Mann-Whitey nonparametric test).

Clinical records were available for full analysis for 14 isolates from patients with CL. Evaluation of these records indicated that patients infected with NO^•^-resistant *L. (L.) amazonensis *or *L. (V.) braziliensis *isolates presented with a larger ulcers (mean ± SD diameter = 43.2 ± 18 mm) than patients who had NO^•^-susceptible parasites (18.0 ± 8 mm, p = 0.01;Figure 3). However, no significant differences between these patient groups were observed in the time from first lesion detection by the patient to the time of clinical evaluation by a physician (mean ± SD = 48 ± 34 days for NO^•^-sresistant versus 32 ± 25 days for NO^•^-susceptible isolates, p = 0.3). Although not significant possibly due to small numbers, 50% of patients with ML, a more severe form of disease, had NO^•^-resistant isolates compared to 31% of the CL patients had NO^•^-resistant isolates. Both isolates from patients with DL were NO^•^-susceptible. There was no significant difference between characteristics of the ulcer at initial presentation, patient age, or size of the Montenegro skin reaction to Leishmania antigen between patients harboring NO^•^-resistant versus NO^•^-susceptible *Leishmania *isolates.

## Discussion

In the current study we demonstrated for the first time that some isolates of both *L. (V.) braziliensis *and *L. (L.) amazonensis *promastigotes are resistant to killing by nitric oxide. Additionally, we showed that the amastigotes from two resistant isolates survived and multiplied better than susceptible isolates in resting human macrophages *in vitro*. Macrophages play a pivotal role in *Leishmania *infection. After phagocytosis, *Leishmania *promastigotes enter a parasitophorous vacuole within which the macrophage can provide a safe haven for the parasite to transform into amastigotes and proliferate in a naïve host [6,31]. In an immune host, macrophages can be activated by inflammatory cytokines to produce toxic metabolites that result in intracellular killing of *Leishmania *[32], or their microbicidal capacity can be dampened or abrogated by suppressive cytokines, leading to disease symptoms [33]. Specifically, TNF-α and IFN-γ elaborated by macrophages or T cells synergize to up-regulate iNOS and the NADH oxidase, with the resultant production of reactive nitrogen intermediates (RNI) and reactive oxygen intermediates (ROI), respectively, that mediate killing of intracellular *Leishmania *[32-36]. Macrophages can alternatively produce IL-10 and TGF-β that inhibit leishmanicidal activity. Both of these cytokines enable the parasite to grow locally and disseminate to distant sites [37-39].

Many prior studies focus on the host immune response during leishmaniasis and the ability of host cells/cytokines to influence the outcome of *Leishmania *infection. During the current study we focused instead on the innate susceptibility of the parasite to leishmanicidal molecules, and their ability to resist to a host microbicidal response. We found that human *L. (V.) braziliensis *and *L. (L.) amazonensis *isolates differ in their innate susceptibility to killing by RNI in vitro, falling into two groups based on their resistance to nitric oxide. Susceptible isolates of both *Leishmania *species were nearly totally killed by 8 mM of acidified NaNO_2_, whereas NO^•^-resistant isolates remained viable even in 16 mM NaNO_2_. These divisions were biologically relevant, in that patients with NO^•^-resistant cutaneous isolates produced significantly larger cutaneous ulcers than NO^•^-susceptible *Leishmania *spp. isolates. Other clinical parameters were not different between NO^•^-resistant and NO^•^-susceptible *Leishmania*, arguing against a spurious association. These clinical data suggest that NO^•^-resistance may lead to more aggressive forms of clinical disease. Differences in the time of storage in liquid nitrogen or in the length of in vitro promastigote cultivation could not explain our observations, since both NO^•^-resistant and NO^•^-susceptible parasites had similar time of storage and were expanded in growth medium only after selection to the present study.

Interestingly, a higher proportion of *L. (L.) amazonensis *isolates than *L. (V.) braziliensis *isolates were NO^•^-resistant (73% versus 18%, respectively). A comparable published study showed that promastigotes and amastigotes of *L. (L.) enriettii *were more sensitive to NO^• ^than *L. (L.) major *[21]. In conjunction with our data, this suggests that there are inter- and intra-species variations in susceptibility to toxic nitrogen products.

Isolate-specific differences in NO^• ^susceptibility are consistent with the observed high degree of DNA polymorphism between isolates of *L. (V.) braziliensis *from several endemic areas of Brazil, documented in literature reports. Techniques used to discern these polymorphic sequences include multilocus enzyme electrophoresis (MLEE) and internal transcribed spacers (ITS) between the small and large subunits of the rRNA gene and polymorphic DNA amplification (RAPD) [40-42]. Utilizing polymorphic DNA amplification (RAPD) we reported DNA polymorphisms in *L. (V.) braziliensis *isolates from Corte de Pedra (CP), Bahia, the same location from which the current patient isolates were derived. In addition to finding polymorphism among the *L. (V.) braziliensis *isolates, we published that certain genotypes are associated with specific forms of leishmaniasis [42]. The current study extends these observations to suggest that there are biological differences between *L. (V.) braziliensis *and *L. (L.) amazonensis *isolates that correlate with the clinical course of disease. Murine resistance to *Leishmania *infections depends at least in part on NO^•^-mediated intracellular killing of parasites through the action of iNOS [type 2 NO^•^-synthase (NOS2)]. However, the contribution of iNOS to parasite killing in human macrophages remains debated. Some reports claim a role for nitric oxide in killing of intracellular *M. tuberculosis *by human alveolar macrophages [18,43]. Our group and another has published evidence for a role of nitric oxide in macrophage microbicidal activity toward *L. (L.) chagasi/infantum *[19,44]. Nonetheless prior studies have reported difficulty in demonstrating NO^• ^production by human macrophages [17].

In addition to NO^• ^derived from the macrophage, the *Leishmania *spp. themselves are able to produce NO^• ^[45-47]. It is likely that the parasite additionally has innate mechanisms for NO^• ^resistance in order to avoid toxicity from endogenous NO^•^. As such, the toxic effects of exogenous NO^• ^generated by macrophages or added experimentally would be expected to represent the sum of NO^• ^generated by the parasite plus exogenous NO^•^, minus the amount of NO^• ^scavenged or inactivated by innate parasite defense mechanisms. We hypothesize that such anti-NO^• ^defenses may be utilized by the parasite for anti-oxidant defense in human infections. This hypothesis is supported by our observation that the degree of NO^• ^resistance correlates with the severity of lesion.

We have previously reported that *L. chagasi *isolates from Brazilians with visceral leishmaniasis are susceptible to killing by NO^• ^[19]. We showed in the current study that *Leishmania *isolates obtained from of humans with CL differ in their susceptibility to NO^•^. NO^•^-resistant *Leishmania *isolates were found to enter macrophages at a similar rate as susceptible strains, but they resisted intracellular killing by 72 to 96 h after infection. The timing of intracellular killing is consistent with the kinetics of iNOS induction, which acts 48–72 hours after infection [19]. We hypothesize that NO^• ^resistance is one of the mechanisms enhancing parasite survival. Alternatively or additionally, NO^•^-resistant parasite isolates could inhibit NO^• ^production by macrophages, or other killing mechanisms such as ROI. Importantly, it has been shown that *M. bovis *inhibits NO^•^-mediated killing by murine macrophages [48], as do *Cryptococcus neoformans *[49], *Trypanosoma cruzi *[50], as well, as *L. (L.) amazonensis *infection [51]. Other studies have reported that amastigote surface enzymes can inhibit NO^• ^production and thereby reduce leishmanicidal activity [52,53]. Furthermore, the LPG-associated kinetoplastid membrane protein 11 has been reported to suppress iNOS activity, because it contains monomethylarginine residues, a structural analog of L-arginine, a known inhibitor of iNOS [31,54].

The meaning of differences in NO^• ^susceptibility amongst different *Leishmania *spp. isolates is not entirely straightforward. The finding that NO^•^-resistant *Leishmania *exhibit improved survival within human macrophages may indicate evasion of iNOS catalyzed toxicity as in murine macrophages and a role for iNOS in control of cutaneous leishmaniasis. Alternatively, since NO^• ^can also play a role in signaling within the infected cell, it is possible that NO^• ^resistant isolates are changing the intracellular signaling, or resistant to alternate microbicidal effector molecules not tested here. It seems likely that NO^• ^resistance may contribute to the apparent increased virulence of these parasites in a human host, based on the differences in severity of the clinical parameters evaluated in the present study (significantly larger lesion size, and trend toward more resistant isolates from ML compared to CL patients). Most certainly, factors other than NO^•^ resistance determine in part the differences in lesion size. Such factors could include the magnitude of parasite inoculum, the host immune response, and the effect of saliva. Nonetheless, it is quite interesting that the NO^•^ resistance correlates with disease severity in our small study. Future studies are needed to better determine the clinical effects of NO^• ^resistance on human infection and response to therapy.

## Conclusion

These data suggest that nitric oxide-resistance of *Leishmania *isolates confers a survival benefit for the parasites inside the macrophage, and possibly exacerbates the clinical course of human leishmaniasis.

## Competing interests

The author(s) declare that they have no competing interests.

## Authors' contributions

AG, EMC and RPA participated equally in the study design, and AG and RPA performed all the parasites experiments.

AG, ARJ, MEW, JLH, LWR and RPA drafted the manuscript

PTGL, JMBP and IC participated in the experiments of human macrophage infection

## Pre-publication history

The pre-publication history for this paper can be accessed here:



## References

[B1] Bittencourt AL, Barral A (1991). Evaluation of the histopathological classifications of American cutaneous and mucocutaneous leishmaniasis. Mem Inst Oswaldo Cruz.

[B2] Louzir H, Melby PC, Ben Salah A, Marrakchi H, Aoun K, Ben Ismail R, Dellagi K (1998). Immunologic determinants of disease evolution in localized cutaneous leishmaniasis due to Leishmania major. J Infect Dis.

[B3] Castes M, Trujillo D, Rojas ME, Fernandez CT, Araya L, Cabrera M, Blackwell J, Convit J (1993). Serum levels of tumor necrosis factor in patients with American cutaneous leishmaniasis. Biol Res.

[B4] Marsden PD (1994). Mucosal leishmaniasis due to Leishmania (Viannia) braziliensis L(V)b in Tres Bracos, Bahia-Brazil. Rev Soc Bras Med Trop.

[B5] Costa JM, Marsden PD, Llanos-Cuentas EA, Netto EM, Carvalho EM, Barral A, Rosa AC, Cuba CC, Magalhaes AV, Barreto AC (1986). Disseminated cutaneous leishmaniasis in a field clinic in Bahia, Brazil: a report of eight cases. J Trop Med Hyg.

[B6] Alexander J, Russell DG (1992). The interaction of Leishmania species with macrophages. Adv Parasitol.

[B7] Alexander J, Satoskar AR, Russell DG (1999). Leishmania species: models of intracellular parasitism. J Cell Sci.

[B8] Liew FY, O'Donnell CA (1993). Immunology of leishmaniasis. Adv Parasitol.

[B9] Scott P (2003). Development and regulation of cell-mediated immunity in experimental leishmaniasis. Immunol Res.

[B10] Wilson ME, Jeronimo SM, Pearson RD (2005). Immunopathogenesis of infection with the visceralizing Leishmania species. Microb Pathog.

[B11] Liew FY, Parkinson C, Millott S, Severn A, Carrier M (1990). Tumour necrosis factor (TNF alpha) in leishmaniasis. I. TNF alpha mediates host protection against cutaneous leishmaniasis. Immunology.

[B12] Liew FY, Wei XQ, Proudfoot L (1997). Cytokines and nitric oxide as effector molecules against parasitic infections. Philos Trans R Soc Lond B Biol Sci.

[B13] Bogdan C, Rollinghoff M, Diefenbach A (2000). The role of nitric oxide in innate immunity. Immunol Rev.

[B14] Green SJ, Crawford RM, Hockmeyer JT, Meltzer MS, Nacy CA (1990). Leishmania major amastigotes initiate the L-arginine-dependent killing mechanism in IFN-gamma-stimulated macrophages by induction of tumor necrosis factor-alpha. J Immunol.

[B15] Evans TG, Thai L, Granger DL, Hibbs JB (1993). Effect of in vivo inhibition of nitric oxide production in murine leishmaniasis. J Immunol.

[B16] Assreuy J, Cunha FQ, Epperlein M, Noronha-Dutra A, O'Donnell CA, Liew FY, Moncada S (1994). Production of nitric oxide and superoxide by activated macrophages and killing of Leishmania major. Eur J Immunol.

[B17] Murray HW, Teitelbaum RF (1992). L-arginine-dependent reactive nitrogen intermediates and the antimicrobial effect of activated human mononuclear phagocytes. J Infect Dis.

[B18] Nicholson S, Bonecini-Almeida Mda G, Lapa e Silva JR, Nathan C, Xie QW, Mumford R, Weidner JR, Calaycay J, Geng J, Boechat N (1996). Inducible nitric oxide synthase in pulmonary alveolar macrophages from patients with tuberculosis. J Exp Med.

[B19] Gantt KR, Goldman TL, McCormick ML, Miller MA, Jeronimo SM, Nascimento ET, Britigan BE, Wilson ME (2001). Oxidative responses of human and murine macrophages during phagocytosis of Leishmania chagasi. J Immunol.

[B20] Bogdan C (2001). Nitric oxide and the immune response. Nat Immunol.

[B21] Mauel J, Ransijn A (1997). Leishmania spp.: mechanisms of toxicity of nitrogen oxidation products. Exp Parasitol.

[B22] Romao PR, Tovar J, Fonseca SG, Moraes RH, Cruz AK, Hothersall JS, Noronha-Dutra AA, Ferreira SH, Cunha FQ (2006). Glutathione and the redox control system trypanothione/trypanothione reductase are involved in the protection of Leishmania spp. against nitrosothiol-induced cytotoxicity. Braz J Med Biol Res.

[B23] Paramchuk WJ, Ismail SO, Bhatia A, Gedamu L (1997). Cloning, characterization and overexpression of two iron superoxide dismutase cDNAs from Leishmania chagasi: role in pathogenesis. Mol Biochem Parasitol.

[B24] Meshnick SR, Eaton JW (1981). Leishmanial superoxide dismutase: a possible target for chemotherapy. Biochem Biophys Res Commun.

[B25] Vickers TJ, Wyllie S, Fairlamb AH (2004). Leishmania major elongation factor 1B complex has trypanothione S-transferase and peroxidase activity. J Biol Chem.

[B26] Levick MP, Tetaud E, Fairlamb AH, Blackwell JM (1998). Identification and characterisation of a functional peroxidoxin from Leishmania major. Mol Biochem Parasitol.

[B27] Miller MA, McGowan SE, Gantt KR, Champion M, Novick SL, Andersen KA, Bacchi CJ, Yarlett N, Britigan BE, Wilson ME (2000). Inducible resistance to oxidant stress in the protozoan Leishmania chagasi. J Biol Chem.

[B28] Fang FC (1997). Perspectives series: host/pathogen interactions. Mechanisms of nitric oxide-related antimicrobial activity. J Clin Invest.

[B29] Cupolillo E, Grimaldi G, Momen H (1994). A general classification of New World Leishmania using numerical zymotaxonomy. Am J Trop Med Hyg.

[B30] Wilson ME, Andersen KA, Britigan BE (1994). Response of Leishmania chagasi promastigotes to oxidant stress. Infect Immun.

[B31] Bogdan C, Gessner A, Solbach W, Rollinghoff M (1996). Invasion, control and persistence of Leishmania parasites. Curr Opin Immunol.

[B32] Murray HW, Nathan CF (1999). Macrophage microbicidal mechanisms in vivo: reactive nitrogen versus oxygen intermediates in the killing of intracellular visceral Leishmania donovani. J Exp Med.

[B33] Reiner NE (1994). Altered cell signaling and mononuclear phagocyte deactivation during intracellular infection. Immunol Today.

[B34] Scott P (1991). IFN-gamma modulates the early development of Th1 and Th2 responses in a murine model of cutaneous leishmaniasis. J Immunol.

[B35] Vouldoukis I, Riveros-Moreno V, Dugas B, Ouaaz F, Becherel P, Debre P, Moncada S, Mossalayi MD (1995). The killing of Leishmania major by human macrophages is mediated by nitric oxide induced after ligation of the Fc epsilon RII/CD23 surface antigen. Proc Natl Acad Sci U S A.

[B36] Kumar P, Pai K, Pandey HP, Sundar S (2002). NADH-oxidase, NADPH-oxidase and myeloperoxidase activity of visceral leishmaniasis patients. J Med Microbiol.

[B37] Bogdan C, Vodovotz Y, Nathan C (1991). Macrophage deactivation by interleukin 10. J Exp Med.

[B38] Barral-Netto M, Badaro R, Barral A, Almeida RP, Santos SB, Badaro F, Pedral-Sampaio D, Carvalho EM, Falcoff E, Falcoff R (1991). Tumor necrosis factor (cachectin) in human visceral leishmaniasis. J Infect Dis.

[B39] Wilson ME, Young BM, Davidson BL, Mente KA, McGowan SE (1998). The importance of TGF-beta in murine visceral leishmaniasis. J Immunol.

[B40] Cupolillo E, Momen H, Grimaldi G (1998). Genetic diversity in natural populations of New World Leishmania. Mem Inst Oswaldo Cruz.

[B41] Cupolilo SM, Souza CS, Abreu-Silva AL, Calabrese KS, Goncalves da Costa SC (2003). Biological behavior of Leishmania (L.) amazonensis isolated from a human diffuse cutaneous leishmaniasis in inbred strains of mice. Histol Histopathol.

[B42] Schriefer A, Schriefer AL, Goes-Neto A, Guimaraes LH, Carvalho LP, Almeida RP, Machado PR, Lessa HA, de Jesus AR, Riley LW, Carvalho EM (2004). Multiclonal Leishmania braziliensis population structure and its clinical implication in a region of endemicity for American tegumentary leishmaniasis. Infect Immun.

[B43] Rockett KA, Brookes R, Udalova I, Vidal V, Hill AV, Kwiatkowski D (1998). 1,25-Dihydroxyvitamin D3 induces nitric oxide synthase and suppresses growth of Mycobacterium tuberculosis in a human macrophage-like cell line. Infect Immun.

[B44] Vouldoukis I, Becherel PA, Riveros-Moreno V, Arock M, da Silva O, Debre P, Mazier D, Mossalayi MD (1997). Interleukin-10 and interleukin-4 inhibit intracellular killing of Leishmania infantum and Leishmania major by human macrophages by decreasing nitric oxide generation. Eur J Immunol.

[B45] Basu NK, Kole L, Ghosh A, Das PK (1997). Isolation of a nitric oxide synthase from the protozoan parasite, Leishmania donovani. FEMS Microbiol Lett.

[B46] Genestra M, Souza WJ, Guedes-Silva D, Machado GM, Cysne-Finkelstein L, Bezerra RJ, Monteiro F, Leon LL (2006). Nitric oxide biosynthesis by Leishmania amazonensis promastigotes containing a high percentage of metacyclic forms. Arch Microbiol.

[B47] Genestra M, Guedes-Silva D, Souza WJ, Cysne-Finkelstein L, Soares-Bezerra RJ, Monteiro FP, Leon LL (2006). Nitric oxide synthase (NOS) characterization in Leishmania amazonensis axenic amastigotes. Arch Med Res.

[B48] Hanano R, Kaufmann SH (1995). Nitric oxide production and mycobacterial growth inhibition by murine alveolar macrophages: the sequence of rIFN-gamma stimulation and Mycobacterium bovis BCG infection determines macrophage activation. Immunol Lett.

[B49] Kawakami K, Zhang T, Qureshi MH, Saito A (1997). Cryptococcus neoformans inhibits nitric oxide production by murine peritoneal macrophages stimulated with interferon-gamma and lipopolysaccharide. Cell Immunol.

[B50] Pakianathan DR, Kuhn RE (1994). Trypanosoma cruzi affects nitric oxide production by murine peritoneal macrophages. J Parasitol.

[B51] Balestieri FM, Queiroz AR, Scavone C, Costa VM, Barral-Netto M, Abrahamsohn Ide A (2002). Leishmania (L.) amazonensis-induced inhibition of nitric oxide synthesis in host macrophages. Microbes Infect.

[B52] Descoteaux A, Turco SJ (1999). Glycoconjugates in Leishmania infectivity. Biochim Biophys Acta.

[B53] Nathan C, Xie QW (1994). Nitric oxide synthases: roles, tolls, and controls. Cell.

[B54] Jardim A, Funk V, Caprioli RM, Olafson RW (1995). Isolation and structural characterization of the Leishmania donovani kinetoplastid membrane protein-11, a major immunoreactive membrane glycoprotein. Biochem J.

